# Anatomical location, sex, and age modulate adipocyte progenitor populations in perivascular adipose tissues

**DOI:** 10.3389/fphys.2024.1411218

**Published:** 2024-07-12

**Authors:** C. Javier Rendon, Lorenzo Sempere, Adam Lauver, Stephanie W. Watts, G. Andres Contreras

**Affiliations:** ^1^ Department of Large Animal Clinical Sciences, College of Veterinary Medicine, Michigan State University, East Lansing, MI, United States; ^2^ Department of Radiology and Precision Health Program, Michigan State University, East Lansing, MI, United States; ^3^ Department of Pharmacology and Toxicology, Michigan State University, East Lansing, MI, United States

**Keywords:** hypertension, adipocyte, remodeling, stem cell, fat, progenitors

## Abstract

Perivascular adipose tissue (PVAT) regulates vascular function due to its capacity to synthesize vasoactive products and its mechanical properties. PVATs most abundant cells are adipocytes, and their populations are maintained by the maturation of adipocyte progenitor cells (APC), which may play a pivotal role in the pathogenesis of cardiovascular diseases. However, the distribution of APC within PVAT depots, their potential variation in spatial location, and the influence of sex and age on their abundance remain unknown. We hypothesize that APC abundance in PVAT is affected by location, age, sex and that APC subtypes have specific spatial distributions. PVAT from thoracic and abdominal aorta, and mesenteric arteries, and AT from interscapular, gonadal, and subcutaneous depots from 13-week and 30-week-old females and males Pdgfrα-CreERT2 x LSL-tdTomato mice (n = 28) were analyzed. Abdominal aorta PVAT had fewer progenitors than mesenteric PVAT and gonadal AT. Aging reduced the abundance of APC in the thoracic aorta but increased their numbers in mesenteric PVAT. Females had more APC than males in mesenteric PVAT and gonadal AT depots. APC exhibited unique spatial distribution in the aorta and mesenteric PVAT where they localized neighboring vasa vasorum and arteries. APC subtypes (APC1, APC2, APC3, diff APC) were identified in all PVAT depots. Thoracic aorta PVAT APC3 were located in the adventitia while diff APC were in the parenchyma. This study identified variability in APC populations based on depot, age, and sex. The distinctive spatial distribution and the presence of diverse APC subtypes suggest that they may contribute differently to cardiovascular diseases-induced PVAT remodeling.

## Graphical Abstract

**Figure:**
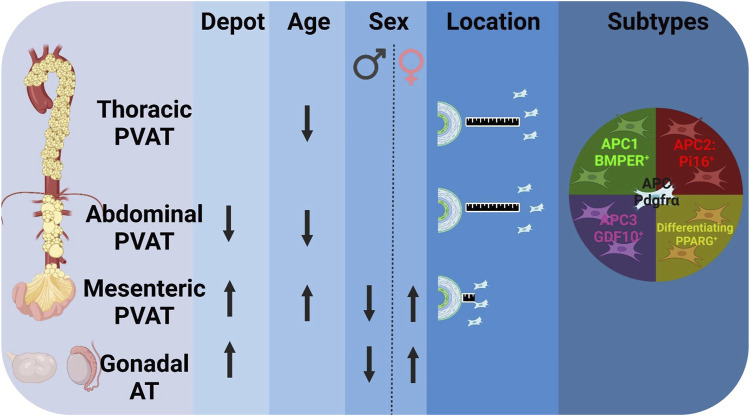
Adipocyte Progenitor Cells (APC) from Perivascular Adipose Tissue (PVAT) is affected by depot, age, sex, and are distributed in different subtypes. Percentage of APC (Pdgfrα+) differs by depot: fewer APC in abdominal PVAT compared to mesenteric PVAT and gonadal adipose tissue (AT). Aging reduced the APC in aortic PVAT, while increased abundance in mesenteric PVAT. Sex differences in mesenteric PVAT and gonadal AT evidence increased APC in females compared to males. More APC (white) were found farther away from aorta in aortic PVAT vs. mesenteric PVAT. APC subtypes are found in all different PVAT depots. (Arrows represent (↑) increase or (↓) decrease abundance of APC (Lin-[CD31-/CD45-]/Pdgfrα+) in total live cells in stromal vascular fraction. BMPER: Bone morphogenic protein endothelial receptor, PI16: Proteinase inhibitor, GDF10: Growth differentiation factor 10, PPARG: Peroxisome proliferator activator receptor gamma.

## 1 Introduction

Hypertension and other cardiovascular diseases alter the structure of conduit (e.g., thoracic and abdominal aorta) and resistance (e.g., mesenteric arteries) vessels (Khavandi et al., 2009; Van Varik et al., 2012; Lima et al., 2019; Nosalski et al., 2020; Wang et al., 2022; Zhuang et al., 2022). This remodeling process encompasses extracellular matrix deposition and the proliferation of diverse cell types in the intima, media, and adventitia tunicas. However, it is less well known if the same processes occur in the tunica adiposa also known as perivascular adipose tissue (PVAT). In other AT, remodeling processes induced by catabolic or anabolic states are driven by the proliferation and differentiation of adipocyte progenitor cells (APC). Understanding the population dynamics of APC in PVAT is important since this tunica regulates blood pressure through the secretion of vasoactive compounds released by its cellular components (mainly adipocytes) and by modulating the biophysics of the vascular structure (Cao et al., 2017; Tuttle et al., 2022).

In general, AT’s adipocyte populations are maintained by adipogenesis of APC. This proliferative population is a subset of stem cells that retain the pluripotent capacity to differentiate into multiple cell types other than adipocytes (Pereira et al., 1995; Krebsbach et al., 1997; Pittenger et al., 1999; Merrick et al., 2019). Platelet-derived growth factor receptor alpha (PDGFRα) is broadly expressed in PVAT and non-PVAT depots and is considered a *bona fide* marker for APC (Lee et al., 2012; Berry and Rodeheffer, 2013; Contreras et al., 2016). Multiple independent research groups have isolated cells expressing PDGFRα and demonstrated that these cells preferentially differentiates into adipocytes in several ATs *in vivo and in vitro* (Lee et al., 2012; Berry and Rodeheffer, 2013; Contreras et al., 2016; Wang et al., 2018; Shin et al., 2020). Specifically, one of these studies demonstrated that transplantation of PDGFRα^+^ cells subcutaneously *in vivo* can contribute 20-fold more to the adipocyte population vs. PDGFRα^−^ cells (Lee et al., 2012). Upon differentiation, APC expresses PPARγ, the key master regulator of adipogenesis that triggers many of the metabolic functions of adipocytes (Tontonoz et al., 1998; Barak et al., 1999). Physiological factors have a strong influence on progenitors’ populations. One of them is sex. Estrogens, for example, promote the proliferation of progenitor cells in female animals (Zhang et al., 2016; Bjune et al., 2022). Age also impacts APC populations with reports indicating a reduction in the proliferation and differentiation capacities of these progenitors as animals get older (Kirkland et al., 1990; Park et al., 2021). However, the effect of sex, age, and PVAT’s anatomical localization on APC remains unknown.

Recent single-cell transcriptomics studies identified APC phenotypic variability (Angueira et al., 2021; Burl et al., 2022; Thompson et al., 2023). In interscapular brown adipocyte tissue (BAT), three APC subtypes were described (Burl et al., 2022). APC1 is characterized by expression of bone morphogenic protein endothelial receptor (*Bmper),* APC2 expressing, proteinase inhibitor 16 (*Pi16)*, and APC3 that expresses growth differentiating factor 10 (*Gdf10).* Notably, APC subtypes express marker genes related to extracellular matrix deposition and cellular phenotype related to PVAT remodeling. In BAT, APC1 preferentially differentiates into adipocytes (Burl et al., 2022; Garritson et al., 2023). APC2 express markers related to TGF-β signaling, a potent fibrogenic pathway, indicating a possible preference for fibrogenic fate (Soare et al., 2020; Ohm et al., 2023). APC3 are associated with osteogenesis differentiation and ossification of carotid arteries (Hino et al., 2012; Brandt et al., 2022). Remarkably, APC subtypes exhibit unique spatial distribution in BAT. APC1 are mainly localized in the parenchyma while APC2 are abundant in the fascia and APC3 neighboring blood vessels. The spatial distribution patterns of APCs and their subtypes in PVAT are unknown. Thus, it is relevant to elucidate how different APC could contribute to PVAT remodeling differently.

This study aimed to determine the presence of APC and their subtypes in different PVAT and AT depots and evaluate the effect of sex, age, and anatomical location on APC distribution. We hypothesize that APC abundance is modified by anatomical location, age, sex and that APC subtypes have specific spatial distributions. Our findings reveal the ubiquitous presence of APCs within PVAT depots and demonstrate their abundance varies across sexes, ages, and depots. Notably, different APC subtypes exhibit distinct spatial distributions within PVAT. These combined observations suggest heterogeneity in the APC response to hypertension, potentially influencing vascular remodeling in a sex-, age-, and anatomically-dependent manner during hypertensive states.

## 2 Materials and methods

### 2.1 Animal models and tissue collections

B6.129S-Pdgfratm1.1 (cre/ERT2)Blh/J (Pdgfrα-CreERT2 mice; stock no. 032770 RRID:IMSR_JAX:032770), and B6.Cg-Gt (ROSA)26Sor_tm9(CAG-tdTomato)Hze/J (LSL-tdTomato; stock no. 007909 RRID:IMSR_JAX:007909) mice were purchased from Jackson Laboratory. All mice were housed at 22°C ± 2°C with a 12:12 h light-dark cycle in an AAALAC-approved animal facility at Michigan State University (East Lansing, MI). Mice were fed a standard chow diet of 18% protein *ad libitum* (Teklad, 2918). Animal protocols were approved by the Institutional Animal Care and Use Committee at Michigan State University (#PROTO202000239) and followed the “Guide for the Care and Use of Laboratory Animals,” 8th edition (Courtney et al., 2021). All mice were euthanized with carbon dioxide administration at a 30%–70% flow rate at 13 or 30 weeks of age, clinical death was confirmed after the mice stopped breathing, the corneal reflex was absent, and no heartbeat could be felt. Final confirmation of death included cervical dislocation. The 13 weeks of age was selected because animals at this age are considered young adults. In humans, this age segment is the early age at which hypertension starts to develop. The 30 weeks of age was selected because animals at this age are considered mid-age adults. In humans, the prevalence of hypertension increases twice after 50 years (mid-age) in both sexes (Flurkey et al., 2007; National Center for Health Statistics, 2018).

### 2.2 Cre recombination induction in transgenic mice

Cre recombination in Pdgfrα-CreERT2 x LSL-tdTomato mice was induced by administering tamoxifen dissolved in corn oil (Sigma, T5648-5G, 30 mg/mL) once per day (150 µg/gr, intraperitoneal) for 5 consecutive days. Littermates without a Pdgfrα-CreERT2 allele (Pdgfrα-CreERT2 −/− mice) were used as controls. To visualize APC, we employed the progeny of Pdgfrα-CreERT2 x LSL-tdTomato mice. Upon tamoxifen induction, Pdgfrα+ cells permanently express the fluorescent protein tdTomato, allowing us to effectively visualize APC populations. Euthanasia and tissue collections were performed 7 days after the first tamoxifen administration, the time when the peak of recombination occurs without affecting the proliferation or differentiation of cells (Donocoff et al., 2020; O’Rourke et al., 2016).

### 2.3 Blood pressure measurements

Blood pressure measurements were made using sphyngomanometry in conscious, restrained, and warmed mice. Measures were taken in healthy animals before any procedures. Animals were acclimated for 3 days before measurements were considered accurate. Animals were restrained using a device to maintain body temperature and keep animal calm. Measurements were made in the CODA system (High Throughput System, Kent Scientific, Torrington, CT, United States). Tail cuff was inflated 15 times (to a pressure of 250 mm Hg with a slow deflation over period of 20 s) with 30-s intervals between inflations. Blood pressure was obtained during each inflation cycle by a volume pressure recording sensor; the final reading was the average of ten inflations. 15 cycles were recorded. Systolic, diastolic, and mean arterial pressure are reported. Once measures were done, animals were returned to their cage.

### 2.4 Adipose tissue collections

Non-PVAT depots including interscapular brown AT (BAT), inguinal subcutaneous AT (SCAT), perigonadal AT (GON), and PVAT depots thoracic aorta (ATPVAT), abdominal aorta (ABPVAT) and mesenteric arteries (MESPVAT), were dissected and then immersed in Krebs-Ringer Bicarbonate Buffer (KRBB) containing NaCl 135 mM; KCl 5 mM; MgSO4 1 mM; KH2PO4 0.4 mM; Glucose 5.5 mM; HEPES 20 mM (pH 7.4) (Teknova, Cat N° H1030) and supplemented with 100 units/mL of penicillin; 100 μg/mL of streptomycin, 0.25 μg/mL of Amphotericin B and 50 μg/mL of Gentamicin. Under a stereo microscope and on a Silastic-coated dish filled with KRBB, BAT was cleansed of adherent white fat subcutaneous tissue; PVAT was dissected from the aorta and mesenteric arteries, and GON was removed from ovaries and testis.

### 2.5 Adipose tissue enzymatic digestions

AT’s stromal vascular fraction (SVF) was isolated as previously described (Contreras et al., 2016). Briefly, AT depots were collected and minced into small fragments (1–3 mm) and then digested for 1 h at 37°C in a rotisserie incubator using 0.5 mg/mL of Liberase™ TL (Roche diagnostics, Cat N° 5401020001) dissolved in Hanks’ balanced salt solution supplemented with 4% BSA (Bovine serum albumin; Fisher, Cat N° BP9706-100) and 10 mM HEPES. Digested material was filtered through 70 μm cell strainers (Corning, Cat N° 22363548) and then centrifuged for 5 min at 300 x g at 4°C to remove the buoyant cells (adipocytes) from the SVF containing APCs. Pellets were resuspended in RBC lysis buffer 1X (Biolegend, Cat N° 420301), incubated at room temperature for 5 min, and then centrifuged for 5 min at 300 x g at 4°C.

### 2.6 Flow cytometry

SVF pellets were resuspended and incubated for 10 min at 4°C with 10 µL of FcR blocking reagent (Miltenyi Biotec, Cat N° 130-092-575) in 90 µL of FACS solution containing 1X Dulbecco’s PBS, 2% FBS, 0.1% sodium azide (ThermoFisher Cat N° 26628-22-8) for non-specific antibody binding. To exclude dead cells, all samples were incubated with Biolegend Zombie NIR™ fixable viability dye (Biolegend Inc, Cat N° 423106; 1:500 in FACS). Next, cells were incubated with a pool of conjugated monoclonal primary antibodies for 30 min (Pacific Blue™ anti-mouse CD45 RRDI: AB_2876534 (1:50); Brilliant Violet 785™ anti-mouse CD31 RRID: AB_2810334 (1:100)). Before conducting the experiments, the optimal concentrations of all primary antibodies were determined through titration. Following primary incubation, cells were washed, fixed with 2% paraformaldehyde in PBS, washed, and resuspended in FACS solution. In each experiment for gating selection, fluorescence minus one (FMO) control was made for all markers used using one sample. UltraComp eBeads™ (ThermoFisher, Cat N° 01-2222-41) beads were used to do single-stained control of each marker. Data acquisition and compensation were performed in Cytek^®^ Aurora System (Cytek Biosciences, Fremont, CA) using the SpectroFlo^®^ software (Cytek Biosciences) and analyzed in FCS express V.7 (*DeNovo* Software, Pasadena, CA). A gate was drawn to allow the exclusion of aggregates/doublets and another to exclude cellular debris. Gating strategies are summarized in Supplementary Figure S1. Briefly, singlet events and live cells were included, while hematopoietic cells (CD45^+^) and endothelial cells (CD31^+^) were excluded. APC were defined as tdTomato+. Flow cytometry results are expressed as the percentage of the specified population relative to the total of live cells.

### 2.7 Immunohistochemistry

Aorta/mesenteric arteries with complete PVAT were cleaned of blood and paraformaldehyde-fixed for 24 h at room temperature, then embedded in paraffin blocks. Tissue sections (5 microns thick) were cut and mounted on Superfrost^®^ Plus microscope slides (Thermo Scientific, Cat N° 12-550-15) dried at 56°C and stored at room temperature (RT) by the MSU Investigative Histopathology laboratory. To de-paraffinize, slides were washed 2 times with Histochoice Clearing Agent (VWR, Cat N° H103) and 4 times with isopropanol (VWR, Cat N° 9084-03) and 2 times with distilled water for 3 min (min) each wash. Antigen retrieval was performed by boiling slides for 30 s in Antigen Unmasking Solution (Vector Laboratories, Cat N° H3301). Slides were rinsed in distilled water and air dried. To contain the blocking serum, primary and secondary antibody solutions, circles were drawn around the sections with an ImmEdge Hydrophobic pen (Vector Laboratories, Cat N° H-4000). Slides were incubated at RT with 1.5% normal goat serum (Vector Laboratories, Cat N° S-1000) in Dulbecco’s phosphate-buffered saline (PBS) (Sigma-Aldrich, Cat N° D8537) blocking solution (BS) for 1 h. Positive control sections were incubated with anti-red fluorescent protein (Rockland Lab, Cat N° 600-401-379; 1:1000) and anti-alpha SMA-FITC (Sigma F3777; 1:500) in 1.5% normal goat serum BS, and negative control sections were incubated with BS overnight at 4°C. Negative controls were included without the addition of primary control in Supplementary Figure S2. The primary antibody and BS were removed from the sections and the slides were rinsed in Dulbecco’s PBS 3 times for 5 min each rinse. The slides were incubated in anti-rabbit secondary antibody AlexaFluor 568 (ThermoFisher, Cat N° A11036; 1:1000) for 1 h at RT. The secondary antibody was removed, and slides were rinsed 3 times with Dulbecco’s PBS for 5 min and allowed to dry thoroughly at RT. Vectashield with DAPI (Vector Laboratories, Cat N° H-1500) was applied to the sections and coverslips mounted to the slides. The slides were allowed to dry and harden at 4°C overnight. Images were acquired at 360, 488, and 544 nm on a Nikon C1 microscope, using a ×20 objective, Nikon DS-Qi1MC camera, and NIS elements BR 4.6 software.

### 2.8 Automated quantification of confocal imaging

Color channels from the immunohistochemistry confocal images were exported using NIS-elements viewer v5.21. After exporting the images to QuPath v0.4.4 software (Courtney et al., 2021), color channels were split and only blue (nuclei) and red (APC) were used for the analysis. Three random regions of interest (ROI) were drawn, and only adipose tissue was included while blood vessels and adventitia were excluded. Blue objects were used for cell detection, and APCs were identified using a single measurement classifier as blue objects co-expressing red signals. Data presented is a percentage of APC in the total number of nuclei per ROI.

### 2.9 Spatial distribution analysis

Confocal images of immunohistochemistry sections were analyzed using PyBioProx v1.0.3 as previously described (Dyer et al., 2020). Briefly, using ImageJ confocal images were converted into RGB format. Then, each color channel was separated. A threshold was applied to the red (APC) and green (SMA/aorta) channels and a mask was created for each color. The new mask was exported to PyBioProx developed for Phyton. The distances from one object in red (APC) and the nearest object in green (SMA) were quantified by the software and then transformed to µm (0.62µm/px). The different distances were divided into 11 bins ranging from the nearest distances (0–21 µm) to the farthest distances (>201 µm). The percentage of APC on each bin was graphed and analyzed using GraphPad Prism v.9.

### 2.10 Multiplex immunohistochemistry (5-plex)

Tissue sections were prepared and stained in the Leica Bond-Rx station as previously described (Sempere et al., 2020). Briefly, tyramide signal amplification stain cycles were performed as followed for each marker after heat-induced epitope retrieval (20 min at 99°C with ER2 solution, EDTA pH = 9): incubation for 30 min with primary antibody, then an incubation with Goat anti-rabbit HRP (Bio-rad, Cat N° STAR124P; dilution) or as appropriate for additional 30 min, followed by tyramide-conjugated fluorochrome (FITC, Rhodamine, DyLight 521, DyLight 594, DyLight 650). Rabbit anti-mouse primary antibodies used were anti-BMPER (1:100; ThermoFisher Cat N° BS-6910R), PI16 (1:100; Novusbio Cat N° NBP1-92254), GDF10 (1:500; ThermoFisher Cat N° BS-5720R), PPARG (1:600; ThermoFisher Cat N° MA5-14889), tdTomato/RFP (1:1000; Rockland Lab Cat N° 600-401-379). Between each stain cycle, after 15 min incubation with 3% H2O2 to inactivate HRP, samples were blocked with Normal rabbit serum (1:20; Jackson Lab, Cat N° 011-000–120) for 30 min each before continuing with the next stain cycle. After staining, slides were counterstained with DAPI nuclear marker for 15 min and immediately mounted and coverslipped with Prolong Gold antifade reagents with DAPI (ThermoFisher, Cat N° P36930) and imaged in the Aperio VERSA scanner (Leica Biosystems).

### 2.11 Co-expression quantification analysis

Multiplex (5-plex) immunofluorescence images were analyzed using the ImageScope x64 software (Leica Biosystems). Aperio Cellular IF Algorithm (Leica Biosystems, Cat N° 23CIFWL) was used for APC subtype classification based on differential marker expression. First, nuclei were identified based on the DAPI fluorescence intensity. Then cell segmentation was performed creating a mask 5 µm away from the nuclei border. We used fluorescence intensity to create a threshold to label positive cells for each channel. APC were defined as tdTomato+, while APC subtypes were defined as follows. APC1: tdTomato+/BMPER+, APC2: tdTomato+/PI16+, APC3: tdTomato+/GDF10, and diff APC (differentiating): tdTomato+/PPARG. Images of the separations of the different channels is depicted in Supplementary Figure S3. The analysis was done in three regions of interest (ROI): adventitia, parenchyma, and fascia (outer layer of PVAT). The average ROI area were parenchyma: 106,943 ± 31,138 µm^2^, fascia: 71,926 ± 45,453 µm^2^, and adventitia: 53,524 ± 3,659 µm^2^.

### 2.12 Statistical analysis

Data is reported as mean ± SEM. Data were analyzed by one- or two-way ANOVA using GraphPad Prism v9 software and JMP pro16. Normality assumptions were assessed using the D’Agostino-Pearson test. Post hoc comparisons were performed using the Kruskall-Wallis test. Statistical significance was set at *p* ≤ 0.05.

## 3 Results

### 3.1 Blood pressure is similar in double transgenic mice regardless of sex and age

Blood pressure measurements of healthy double transgenic Pdgfra-Cre-LSL-tdTomato are shown in Table 1. Mean arterial blood pressure (MAP) between females and males at 13- and 30-weeks of age did not differ.

**TABLE 1:** Mean arterial blood pressure (MAP), systolic blood pressure (SBP), and diastolic blood pressure (DBP) of Pdgfra-Cre-LSL-tdTomato mice at 13- and 30-week time points. Two-way ANOVA and Bonferroni test were used. n = 6 for each group.

Groups (n = 6)	MAP (mm Hg)	SBP (mm Hg)	DBP (mm Hg)
**Males 13-weeks**	85.22 ± 4.9	101 ± 5.001	77.73 ± 4.9
**Females 13-weeks**	89.86 ± 4.3	109.7 ± 4.5	80.4 ± 3.3
**Males 30 weeks**	94.10 ± 10.2	104.7 ± 4.5	77.91 ± 4.5
**Females 30 weeks**	97.69 ± 10.5	109.5 ± 3.4	81.60 ± 4.93

### 3.2 APC are present in PVAT, and their abundance varies by anatomical location

**FIGURE 1:**
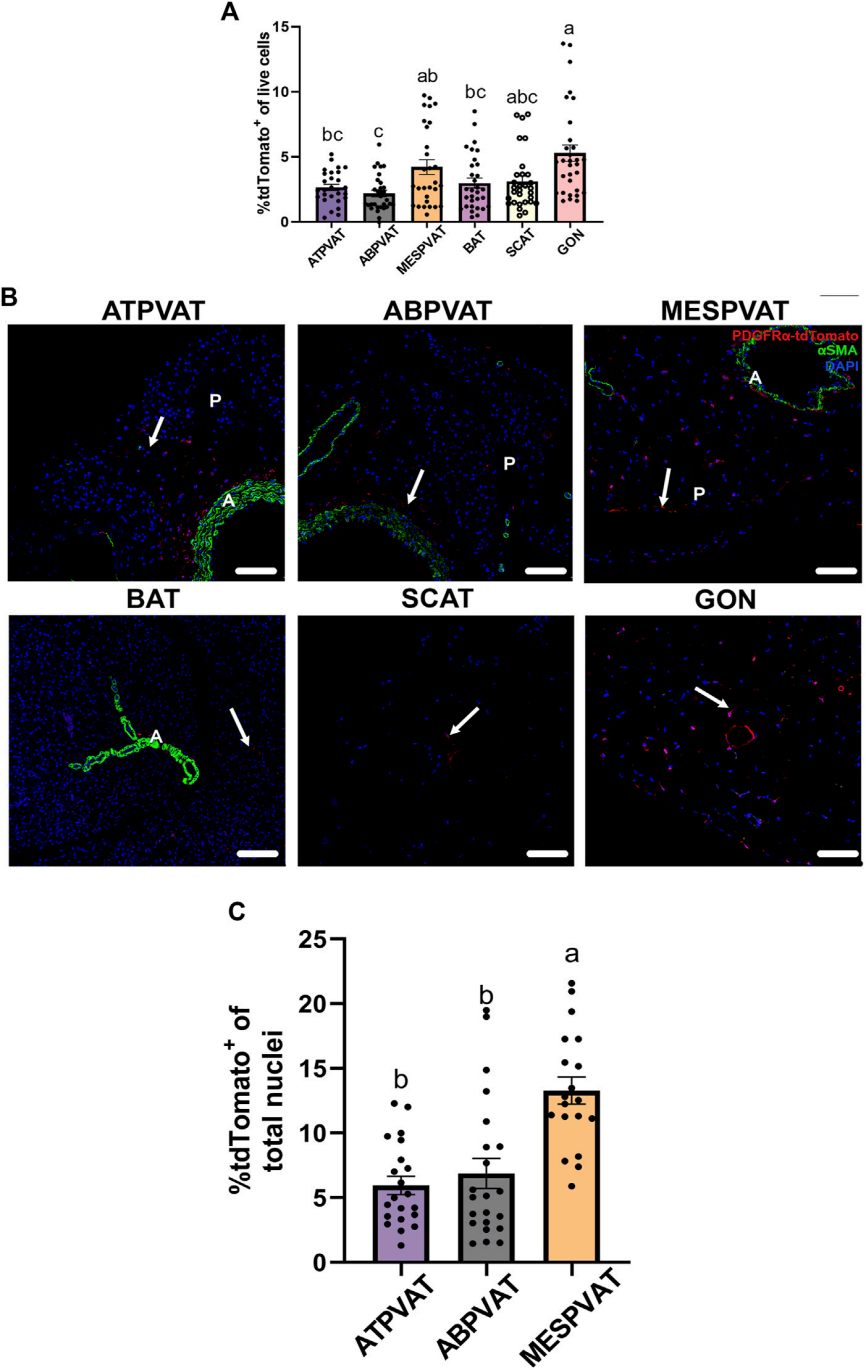
Adipocyte progenitor cells (APC) are present in PVAT. **(A)** Adipose tissue (AT) was processed for flow cytometry analysis. Difference in frequency of APCs of total live cells (100%) between AT depots of both males and females at 13-weeks and 30-weeks-old. Data are means ± SEM. One-way ANOVA and *t*-test with Kruskal-Wallis’s correction were used. Significant differences between depots are indicated by different letters a, b, and c (*p* < 0.05). **(B)** Representative images (confocal) of AT sections from PdgfrαCre-LSL-tdTomato mice including non-perivascular AT interscapular (BAT), subcutaneous (SCAT), perigonadal (GON) AT and perivascular AT from thoracic aorta (ATPVAT), abdominal aorta (ABPVAT) and mesenteric arteries (MESPVAT). Antibodies against Red Fluorescent Protein (RFP/tdTomato), and alpha smooth muscle actin (αSMA) were used. Red = APC/tdTomato, green: αSMA. DAPI was used as counterstain nuclei. Arrow shows APC (tdTomato+). n = 30 mice were used. Scale bar: 100 µm (P= Parenchyma, L = Lumen). **(C)** Quantification of confocal images of PVAT sections. APC (tdTomato+) percentage of total nuclei (DAPI) per region of interest (ROI). Three random areas were selected. Data are means ± SEM. One-way ANOVA with the Kruskal-Wallis’s correction test was used. Significant differences between each depot are indicated by different letters a and b (*p* < 0.05).

Flow cytometry analysis demonstrated that the percentage of APC in SVF from different AT varied by anatomical location, including PVAT depots. Combined data from both sexes and ages demonstrated that in ATPVAT, APC were 2.86% ± 0.31 of the SVF population. In other PVAT depots, APC were 2.18% ± 0.24 of SVF in ABPVAT and 4.71% ± 0.51 in MESPVAT. In non-PVAT depots: BAT had 2.99% ± 0.38 of APC, SCAT 2.55% ± 0.28 and GON 4.71% ± 0.51. Comparing the abundance among all different depots, we found that ABPVAT had fewer APC in total live cells than MESPVAT and GON depots (Figure 1A). To confirm the presence of APC in the different depots we used confocal microscopy to identify PDGFRα expressing cells (tdTomato+). In all depots regardless of their location, we observed APCs (Figure 1B). Quantification of the abundance of APC from the total nuclei in the confocal images from PVAT depots demonstrated that APC were more abundant in MESPVAT (13.28% of total nuclei ± 1.03) vs. ATPVAT (5.94% ± 0.7) and ABPVAT (6.8% ± 1.16) (Figure 1C).

### 3.3 Aging reduces APC abundance in PVAT around aorta

**FIGURE 2:**
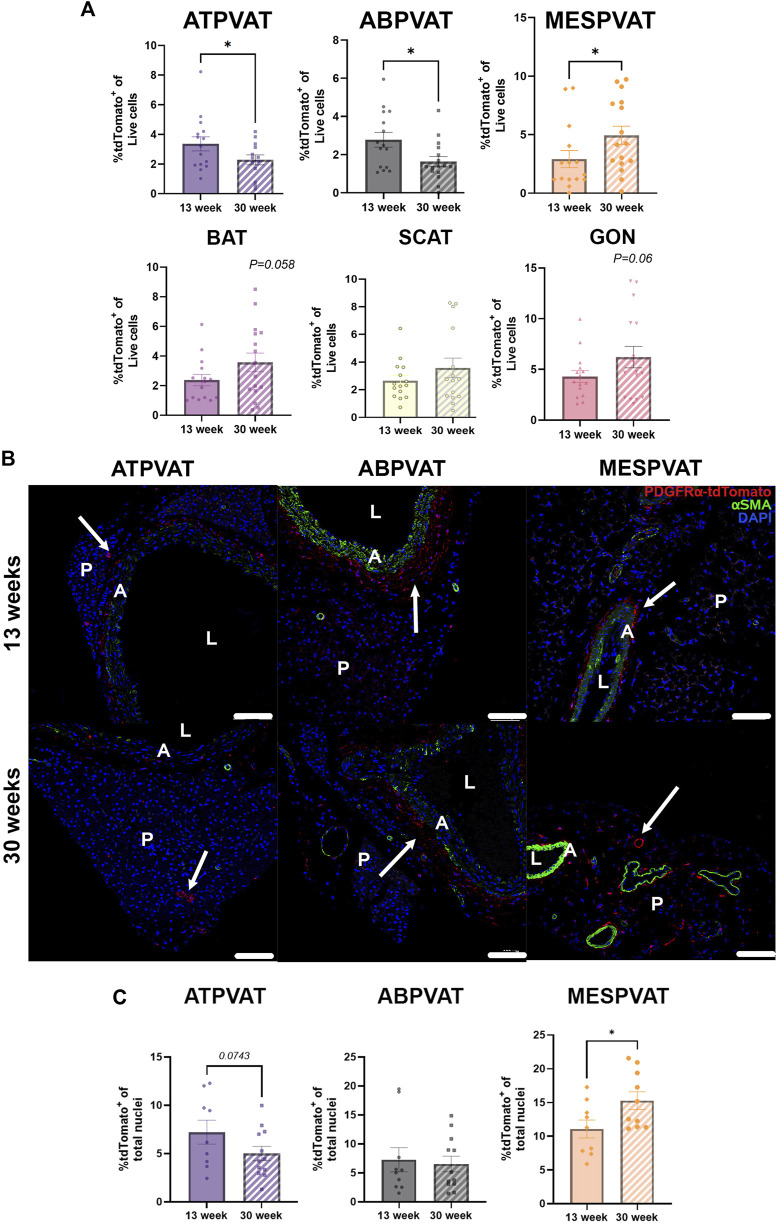
Aging reduces adipocyte progenitor cells (APC) populations aortic perivascular adipose tissues (PVAT). **(A)** Frequencies of APC (P1/Singlets/Live/Lin^−^(CD45^-^/CD31^-^)/tdTomato^+^) in total live cells from perivascular adipose tissues (PVAT) in thoracic aorta (ATPVAT), abdominal aorta (ABPVAT), mesenteric arteries (MESPVAT) and non-PVAT interscapular (BAT), subcutaneous (SCAT), and perigonadal (GON) depots from 13-week and 30-week-old mice. Data are means ± SEM. One-way ANOVA and *t*-test with Kruskal-Wallis’s correction were used. Significant differences between ages on each depot are indicated by *(*p* < 0.05). **(B)** Representative images of ATPVAT, ABPVAT and MESPVAT. APC in red, alpha smooth muscle actin (αSMA) in green, and nuclear stain DAPI blue. Arrows shows APC (tdTomato^+^). n = 15 at 13-weeks and n = 16 at 30-weeks were used. Scale bar: 100 µm (A = aorta/arteries, L = Lumen, P= PVAT). **(C)** Quantification of confocal images of PVAT sections. APC (tdTomato^+^) percentage of total nuclei (DAPI) per region of interest (ROI). Data are means ± SEM. *t*-test with Welch’s correction was used. Significant differences between ages on each depot are indicated by *(*p* < 0.05).

Next, we evaluated the effect of aging on APC populations. In ATPVAT, 30-week-old animals had lower abundance of APCs (2.28% ± 0.33) compared to 13-week-old animals (3.36% ± 0.47) (*p* < 0.05). A similar response was observed in the ABPVAT with 30-week-old mice having fewer APCs (1.63% ± 0.26) vs. 13-week-old (2.77% ± 0.38) (*p* < 0.05). In contrast, MESPVAT from 30-week-old animals had higher % of APC (4.93% ± 0.78) compared to 13-week-old mice (2.91% ± 0.73) (*p* < 0.05). Finally, no differences in APC abundance were observed in non-PVAT depots by age. (Figure 2A). Confocal imaging of PVAT depots reflected flow cytometry results, we observed fewer APCs in ATPVAT and ABPVAT from 30-week-old than 13-week-old mice. In contrast, APC populations increased with age in MESPVAT. (Figure 2B). Image analysis showed a tendency for less APC in ATPVAT between 13 weeks old (7.2% ± 1.2) vs. 30 weeks old (5% ± 0.77) (*p* = 0.07). On the other hand, more APC were found in the MESPVAT from 30-week animals (15.29% ± 1.32) vs. 13-week animals (11.06% ± 1.32) (*p* < 0.05) (Figure 2C).

### 3.4 Mesenteric PVAT and gonadal APC populations exhibit sexual dimorphism in 30-week-old mice

**FIGURE 3:**
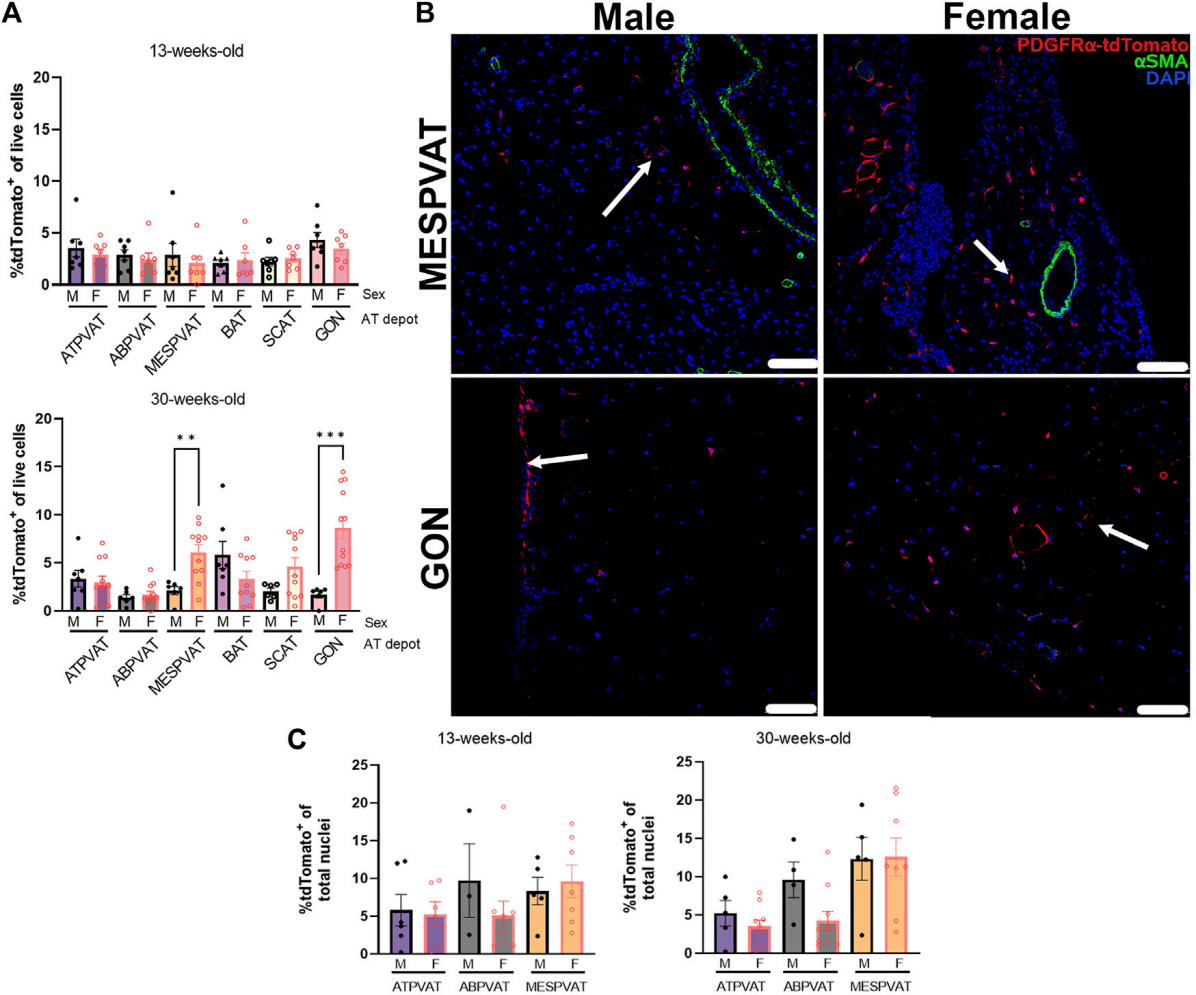
Sex differences in APCs populations appear in white AT depots at 30 weeks of age. **(A)** APC (tdTomato^+^) in total live cells from thoracic aortic perivascular adipose tissue (ATPVAT), abdominal aortic PVAT (ABPVAT), mesenteric arteries (MESPVAT), interscapular (BAT), inguinal subcutaneous (SCAT), and perigonadal (GON) depots from males (M) and females (F) of 13-week (top) and 30-week-old (bottom). Data are means ± SEM. Two-way ANOVA and *t*-test with Kruskal-Wallis’s correction were used. Significant differences between sexes within each depot are indicated by ** (*p* < 0.01) and *** (*p* < 0.001). **(B)** Representative confocal images of mesenteric (MESPVAT) and perigonadal (GON) adipose tissues comparing 30-week-old male and female mice. TdTomato in red, alpha smooth muscle actin (αSMA) in green, and nuclear stain DAPI blue. Arrow shows APC (tdTomato^+^). n = 7 male and n = 7 females in 13-weeks group, and n = 7 male and n = 11 females in 30-weeks group were used. Scale bar: 100 µm. **(C)** Quantification of confocal images of PVAT sections. APC (tdTomato^+^) percentage of total nuclei (DAPI) per region of interest (ROI). Data are means ± SEM. Two-way ANOVA and *t*-test with Welch’s correction were used.

The effect of sex on APC populations was evaluated on each depot and age group separately. Thirteen-week-old mice did not show differences by sex on PVAT and other AT depots. However, in thirty-week-old animals, sex had a strong effect on MESPVAT and GON depots. MESPVAT from females had more APCs (6.07% ± 0.82) compared to males (2.13% ± 0.42). There were more APCs (8.64% ± 1.16) in GON from females vs. males (1.69% ± 0.35; Figure 3A). However, quantification of APC in confocal microscopy images showed no differences between the sexes (Figures 3B,C).

### 3.5 Mesenteric PVAT APCs are more abundant in proximity to blood vessels compared to other PVAT depots

**FIGURE 4:**
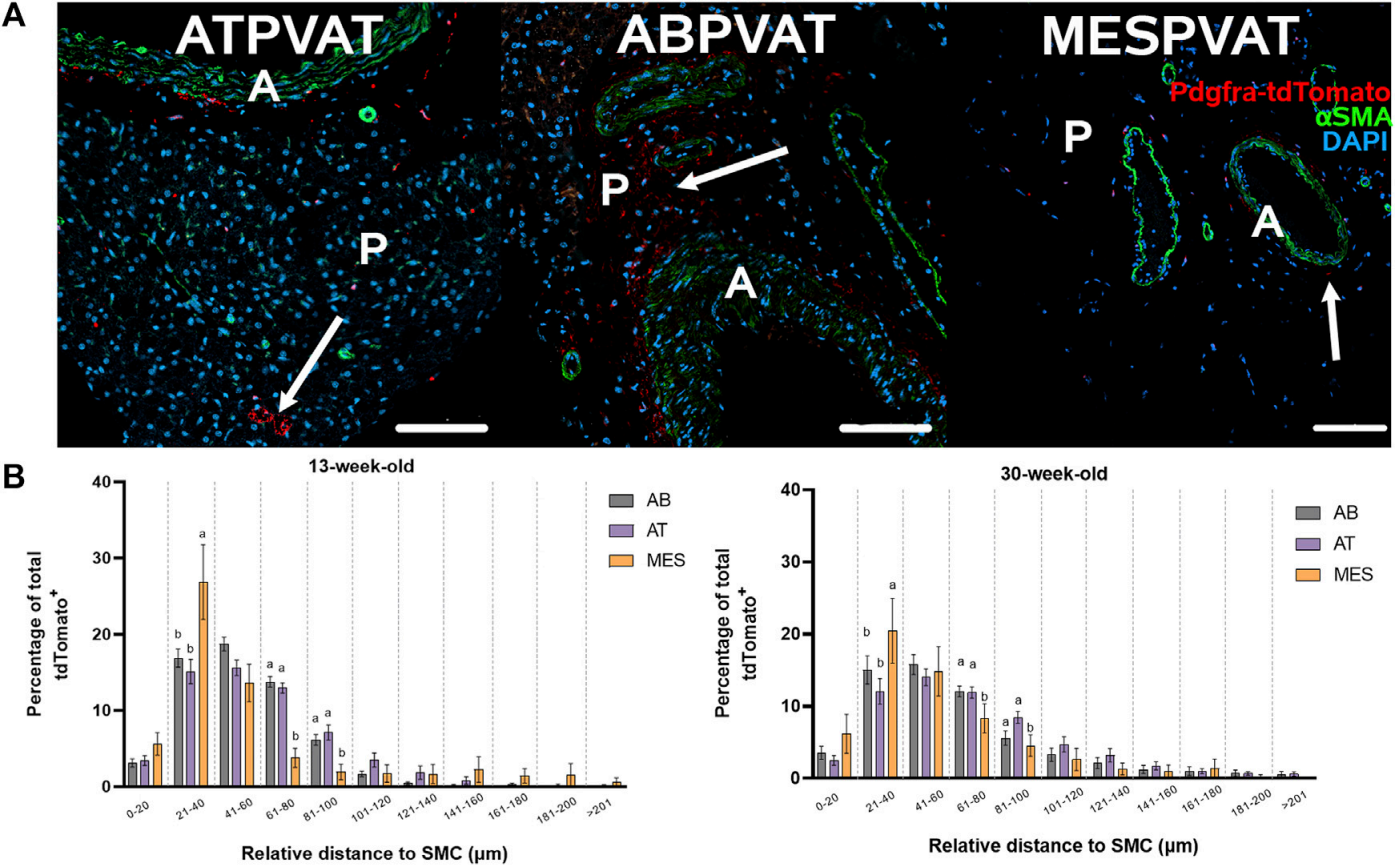
APC abundance increases near blood vessels in mesPVAT compared to other PVATs. **(A)** Representative images of thoracic aortic PVAT (ATPVAT), abdominal aorta PVAT (ABPVAT), and mesenteric arteries (MESPVAT). tdTomato in red, alpha smooth muscle actin (αSMA) in green, and nuclear stain DAPI in blue. Scale bar: 100 µm. Arrows shows APC. **(B)** Distances were measured using PyBioProx on Python. Data is a percentage of APC by bins (separated by a vertical dotted line) of distances relative to the aorta or mesenteric arteries in 13-weeks-old and 30-weeks-old animals. AB: ABPVAT, AT: ATPBAT, and MES: MESPVAT. Data are means ± SEM. Two-way ANOVA and *t*-test with Welch’s correction were used. n = 7 AT, n = 8 AB, and n = 6 MES, animals were used for both age groups. Significant differences between depots on each bin are indicated by different letters a and b (*p* < 0.05).

The spatial distribution of APCs was evaluated using confocal images from ATPVAT, ABPVAT, and MESPVAT (Figure 4A). The proximity of APC to the nearest blood vessel (stained with alpha-actin SMA) was determined using PyBioProx (Dyer et al., 2020). Near blood vessels (21–40 µm), APCs were more abundant in MESPVAT (26.85% ± 4.9) compared ATPVAT (15.11% ± 1.59) and ABPVAT (16.89% ± 1.19; *p* < 0.05) in both 13 and 30-weeks old mice. At more distant locations (61–80 µm), MESPVAT had fewer APC (3.78% ± 1.26) compared to ATPVAT (12.96% ± 0.65) and ABPVAT (13.76% ± 0.68) in 13-week-old mice. Similarly, in 30-week-old mice, there were fewer APC in MESPVAT (8.32% ± 2.05) compared to ATPVAT (11.91% ± 0.80) and ABPVAT (12.06% ± 0.74) (Figure 4B).

### 3.6 PVAT harbors APC subtypes regardless of sex and age

**FIGURE 5:**
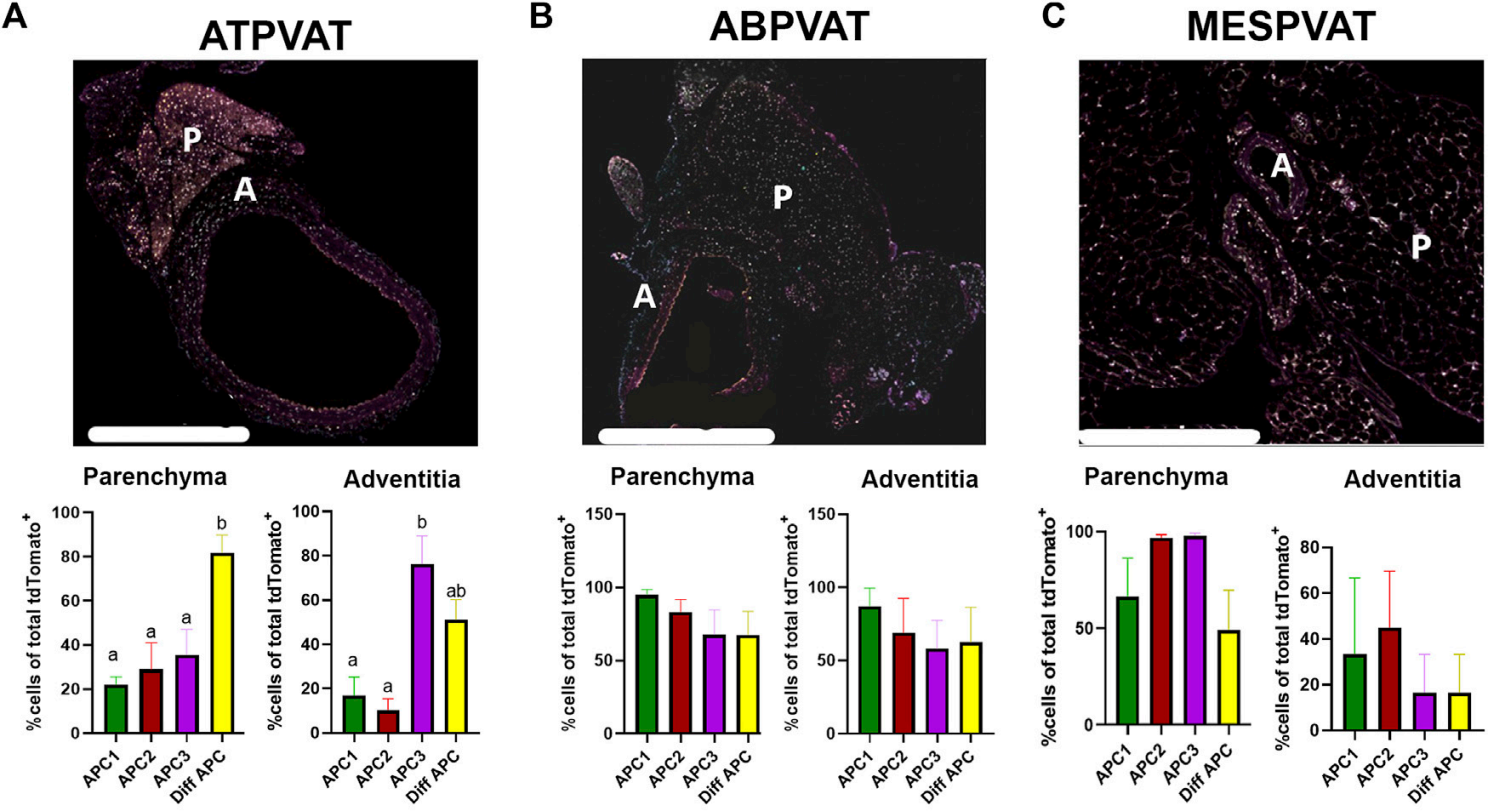
APC subtypes are present in PVAT in both sexes and age groups. 4-plex immunohistochemistry of **(A)** Thoracic PVAT (ATPVAT) **(B)** Abdominal PVAT (ABPVAT) and **(C)** Mesenteric arteries (MESPVAT) (Regions of interest in dotted line indicating P: Parenchyma, A: adventitia). Each depot had an analysis of the frequency of APCs of total tdTomato APCs (100%) on each subtype (APC1: BMPER, APC2: PI16, APC3 GDF10, and Diff APC: PPARG). Regions of interest (ROI): parenchyma and fascia were analyzed. Data are means ± SEM. One-way ANOVA with Welch’s correction was used. Significant differences between APC subtypes on each ROI are indicated by letters a and b (*p* < 0.05). n = 3 males and n = 3 females were analyzed for each age group. Scale bar: 200 µm.

ATPVAT (Figure 5A), ABPVAT (Figure 5B), and MESPVAT (Figure 5C) depots were stained with antibodies to identify APC1, APC2, APC3, and differentiating APC subtypes. Within PVATs, parenchyma and the adventitia were analyzed for APC abundance. Diff APCs were the most abundant populations (81.85% ± 8.001) in ATPVAT parenchyma while in the adventitia, the most prevalent APC subtype was APC3 (76.23% ± 12.7) (Figure 5A). In the ABPVAT, all APC subtypes were found in the parenchyma and adventitia without differences in their spatial distribution (Figure 5B). Finally, in MESPVAT parenchyma we observed that APC2 (96.84% ± 1.5) and APC3 (97.81% ± 1.4) were the most prevalent APC. No differences between APC subtypes were observed in the adventitia (Figure 5C).

## 4 Discussion

### 4.1 Blood pressure was similar between sex and age

Our data demonstrate that blood pressure in double transgenic Pdgfra-Cre-LSL-tdTomato was not affected by sex and there were no differences between 13-week and 30-week-old mice. The systolic blood pressure in our study ranged from 95 to 127 mm Hg, values that can be considered normotensive [100–120 mm Hg (Ichihara et al., 2001; Ciuceis et al., 2005; Zhang et al., 2010; Lu et al., 2015),]. Interestingly, previous studies have found differences in blood pressure that vary depending on genetic backgrounds. Males C57BL/6J have higher systolic BP than females at 21 weeks of age, but these responses were not observed in FVB/N mice (Barsha et al., 2016). Our results demonstrate that there are no differences in blood pressure between sexes at two time points evaluated and that the responses of APC are not due to differences in blood pressure.

### 4.2 The effect of anatomical location on APC pools

Here we identified APC in PVAT and non-PVAT depots. Flow cytometry analyses indicate APC populations ranged from 0.5% to 13% of SVFs. Previous studies in rodents reported similar APC abundances ranging from 1.5% to 14% (Berry and Rodeheffer, 2013; Lee et al., 2013; Contreras et al., 2016; Angueira et al., 2021; van Kuijk et al., 2023). In non-PVAT depots such as GON and SCAT the abundance of APC is around 3% (Lee et al., 2013), in mammary AT between 2% and 5.2% (Wang et al., 2018), and in intermuscular AT around 10% of the SVF (Uezumi et al., 2010). Regarding PVAT depots, our group reported that PDGFRα^+^ cells comprise 1.5% of SVF from ATPVAT and 3% of the same cellular fraction in MESPVAT (Contreras et al., 2016). Seale et al. established that APC populations account for 5% of cells in SVF from ATPVAT. In contrast, Sluimer and colleagues reported 14% APC in SVF from ATPVAT and 8% from ABPVAT (van Kuijk et al., 2023). Different factors could contribute to the variability among studies. First, the markers used to identify progenitors were different among studies. Previous reports have used stem cell antigen 1, CD34, and/or CD24. Here, we defined APC as PDGFRα because it is a marker of committed preadipocytes and can be found across PVAT and non-PVAT depots (Contreras et al., 2016). Second, ages were different among studies, ranging from 8 to 30 weeks old. Additionally, previous studies did not evaluate sex differences, some used only males (Berry and Rodeheffer, 2013; van Kuijk et al., 2023) or combined data from males and females (Lee et al., 2013; Angueira et al., 2021). Third, flow cytometry commonly uses antibodies to detect PDGFRα on the cell surface. When using enzymatic digestions, the structure of many surface proteins can be affected, possibly reducing the sensitivity and specificity of the antibody/antigen binding. To address the latter, we used the lineage tracing model and based APC quantification on the reporter signal (td-tomato) which confers a better approach to quantifying these populations after enzymatic tissue processing.

In the present study, APC abundance showed differences in an AT depot-dependent manner, with GON having more and ABPVAT having fewer APCs (Figure 1). A smaller APC population in ABPVAT could lead to reduced expansion capacity by hyperplasia and favor hypertrophy in this depot. In metabolic diseases limited preadipocyte hyperplasia is being associated with larger adipocytes and pro-inflammatory environment, changes more prevalent in abdominal PVAT (Strissel et al., 2007; Nishimura et al., 2008; Police et al., 2009; Muir et al., 2016). However, further research is needed to evaluate if low APC abundance affects PVAT remodeling during hypertension and other cardiovascular diseases.

### 4.3 Effects of aging on APC abundance in PVAT

Aging reduces the abundance of stem progenitor cells in tissues (Lukjanenko et al., 2019; Shcherbina et al., 2020; Von Bank et al., 2021). Previous reports evaluated the proportions of cells in PVAT with common markers of progenitors, including Sca-1^+^/CD34^+^, during aging. Pan and colleagues found no differences in stem cell abundance with aging in PVAT but reported a loss in their brown adipogenic potential (Pan et al., 2019). In the present study, we demonstrated that APCs were reduced in PVAT around the thoracic and abdominal aorta with aging (Figure 2A). Different mechanisms may explain these effects such as reduced proliferation, decreased viability of progenitors, or loss of self-renewal activity (Kirkland et al., 1990; Oh et al., 2014; Park et al., 2021). Fewer APCs may limit PVAT’s adipocyte turnover, which can reduce the secretion of anticontractile factors, consequently promoting higher vascular stiffness, an outcome observed in aged rodents (Zemančíková and Török, 2019). On the other hand, the increased pool of APCs in MESPVAT could favor adipocyte turnover, potentially increasing anticontractile factors and leading to reduced vascular stiffness. Taken together, these findings may partially explain why arterial stiffness progressively increases with age only in elastic arteries, such as the aorta, and not in muscular arteries like the mesenteric arteries (Ruitenbeek et al., 2008; Borlotti et al., 2012; Zhang et al., 2013; Leloup et al., 2015). This study further supports the established link between age and increased risk of developing hypertension (Sun, 2015; Kohler et al., 2022).

### 4.4 Sex dimorphism and age effects in MESPVAT and GON

Results from the present study indicate that the visceral depots MESPVAT and GON have higher APC abundance in 30-week-old compared to 13-week-old females (Figure 3). These differences may be attributed to age-related sex hormone dynamics. Female mice exhibit an early reproductive stage beginning around 2–3 months (13 weeks), then a reproductive peak around 6 months (26 weeks), and a decline after 13 months (56 weeks) (Nelson et al., 1981; Nelson et al., 1982; Lamberts et al., 1997). The reproductive peak correlates here with higher abundance of APC in 30-week-old female mice. A possible explanation could be the proliferative effect of estrogen on progenitor cells (Lapid et al., 2014; Zhang et al., 2016; Bjune et al., 2022). In humans, APC population dynamics change similarly in terms of sex and age. In subcutaneous abdominal adipose depot of 29 ± 2-year-old females, the APC abundance is 10% higher when compared to age-matched males (Tchoukalova et al., 2010). On the other hand, aging promotes a shift in fat distribution characteristic of an increased expansion of visceral vs. subcutaneous depots (Kuk et al., 2009; Ou et al., 2022). It is important to note that the present study did not include animals older than 7 months. However, our results provide evidence for the impact steroid hormones may have on APC. Future studies will need to address how sex differences may alter APC proliferations and adipogenesis during PVAT remodeling induced by hypertension, and how APC abundance change by depot in senescent animals (13–18 months).

### 4.5 APC are strategically located proximal to blood vessel’s adventitia

Our results show that APCs are closely associated with blood vessels in MESPVAT (<60 μm; Figure 4). This is a common spatial distribution observed in PDGFRα^+^ cells (Wang et al., 2019). However, in ATPVAT and ABPVAT, more APC were located 60 µm away or more within the parenchyma. This spatial distribution pattern was more prominent in 30-week-old animals suggesting that adipogenesis may occur in the adventitia but also in the parenchyma of aortic PVAT in older adults. Since APCs still have the potential to differentiate into cells other than adipocytes and considering that most of them are in proximity to blood vessels in MESPVAT, this may suggest that the remodeling process starts in this area. Further experiments will need to address how hypertension modulates the fate of APCs that are closer to the blood vessels vs. those located within the parenchyma of PVAT.

### 4.6 APC subtypes are found in PVAT and have different spatial distribution

Previous studies identified APC subtypes in both ATPVAT and non-PVAT depots and associated these populations with specific phenotypic fates such as fibrogenesis and adipogenesis (Angueira et al., 2021; Burl et al., 2022). For instance, *Bmper*, expressed in APC1, is a positive regulator of adipogenesis and mediates the cold-induced maturation of new adipocytes that occurs in BAT (Burl et al., 2022; Garritson et al., 2023). APC2 (Pi16 populations) co-express *Dpp4*, a modulator of the TGF-β profibrogenic pathway (Soare et al., 2020; Ohm et al., 2023), while APC3 express *Gdf10*, a mediator of TGF-β and ossification of carotid arteries (Hino et al., 2012; Brandt et al., 2022). In the parenchyma of ATPVAT, we observed high numbers of Diff APC; in contrast, we quantified higher expression of APC3 in the adventitia. This spatial distribution may indicate that regions such as adventitia may be predisposed to have ossification and fibrogenesis, while the parenchyma is more likely to be more prone to adipogenesis. In the rest of the PVAT depots we did not observe differences in APC spatial distribution. Interestingly, some APC co-expressed different markers simultaneously. Although conjecture, these results indicate that the PVAT remodeling process may occur in specific areas within the PVAT. Future experiments need to address how each APC subtype contributes differently to adipogenic and fibrogenic fates, the synthesis of ECM proteins, and the regional plasticity of PVAT remodeling during the development of hypertension.

### 4.7 Physiological relevance

The goal of this study was to establish how APC subpopulations in different PVAT depots, such as the aorta (conduit) and mesenteric arteries (resistant) during normotensive conditions compare to other visceral non-PVAT adipose depots. Our data demonstrate the presence of APC in all PVAT depots. For the first time, we showed uniquely defined spatial distribution of APC subtypes in ATPVAT. We also determined that females had higher APC abundance in visceral depots (MESPVAT and GON), and that age reduces APC abundance in aortic PVAT (ATPVAT and ABPVAT) but increases it in MESPVAT mesenteric depots. The unique APC distribution, and their different subtypes with diverse spatial locations indicate that they contribute differently to hypertension-induced remodeling. The consequences of unique APC distribution and the effects of age and sex in PVAT need to be studied in detail in hypertension animal models and in human patients. Understanding the dynamic role that APC play in the remodeling process of the PVAT during hypertension will contribute to identify new therapeutic targets to treat this disease.

## Data Availability

The raw data supporting the conclusions of this article will be made available by the authors, without undue reservation.
