# Supplementary material for: Anatomical location, sex, and age modulate adipocyte progenitor populations in perivascular adipose tissues
Source: Front Physiol. 2024 Jul 12;15:1411218. doi: 10.3389/fphys.2024.1411218 (PMC11282503; doi:10.3389/fphys.2024.1411218)
Supplement: Supplementary file 1 [file DataSheet1.docx]

Supplementary Material

# Supplementary Data
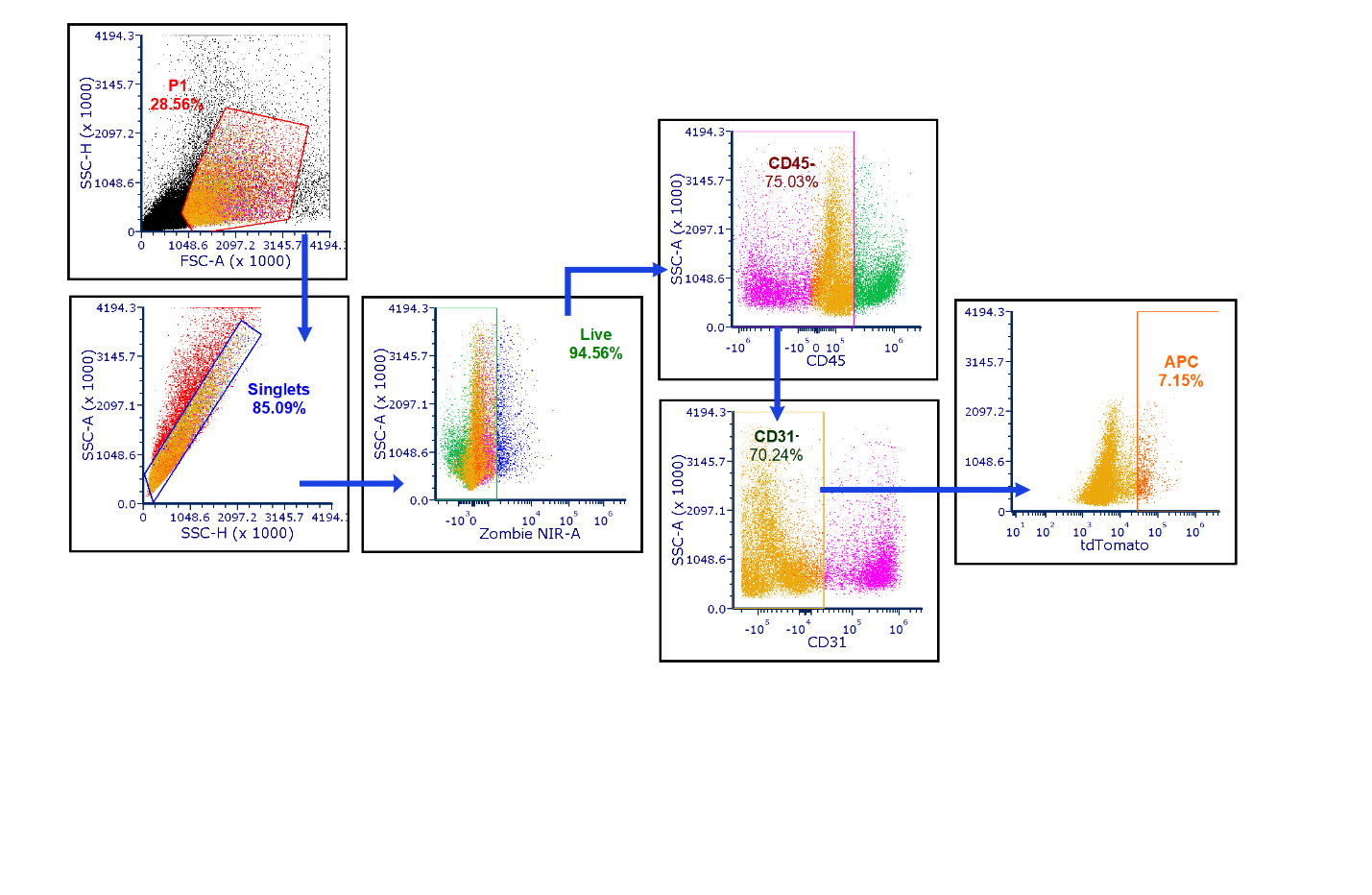


**Supplemental Figure 1.** Flow cytometry gating strategy defining cell populations from adipose tissues from Pdgfra-Cre-LSL-tdTomato mice. The gating strategy flow: A) Inclusion of cells without debris, and B) singlets events. C) Exclusion of dead cell with fixable viability dye, and D) CD45^+^, E) CD31^+^ cells. Finally, APC were identified as F) inclusion of tdTomato^+^.


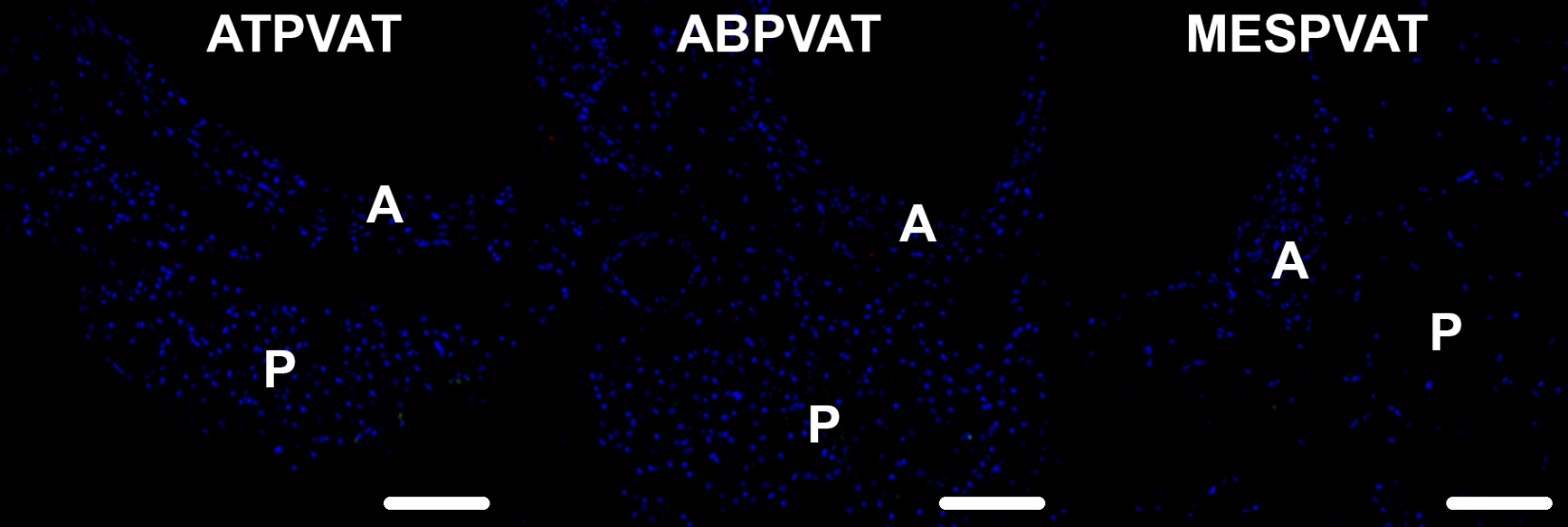


**Supplemental Figure 2.** Representative images of immunofluorescence negative primary antibody controls in A) thoracic (ATPVAT), B) abdominal (ABPVAT), and C) mesenteric (MESPVAT) sections. A cocktail of secondary antibodies was only included with nuclear stain DAPI blue. (A= aorta/arteries, P= PVAT). Scale bar: 200µm.


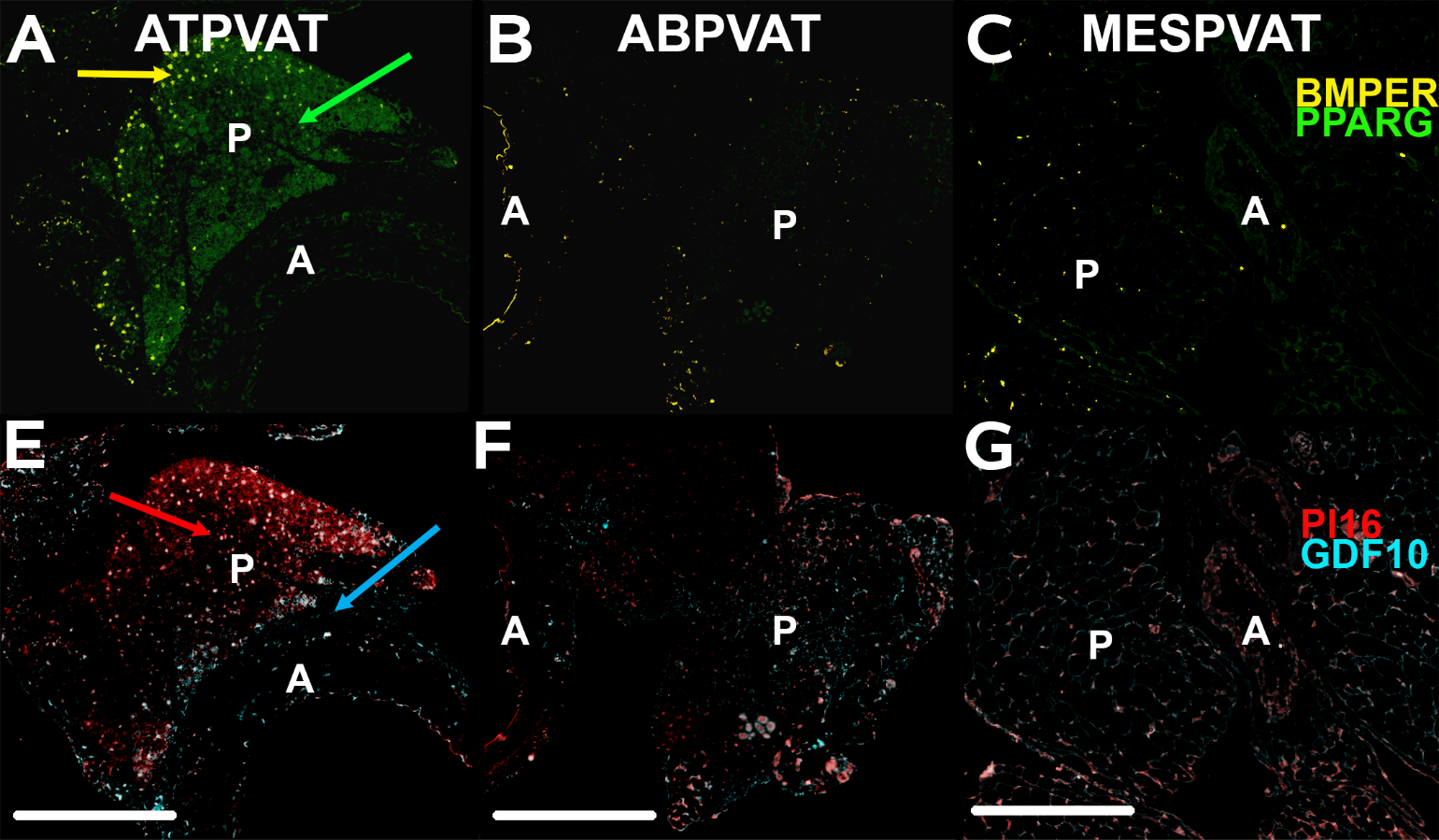


**Supplemental Figure 3.** 4-plex immunohistochemistry of BMPER in the yellow arrow, while PPARγ in green arrows of A) thoracic PVAT (ATPVAT), B) abdominal PVAT (ABPVAT), and C) mesenteric arteries (MESPVAT). Signals of PI16 in red arrows, while GDF10 in cyan are depicted for D) ATPVAT, E) ABPVAT, and F MESPVAT. (P: PVAT, A: Adventitia). Scale bar: 200µm.
